# Case Report: SVF after chemoradiotherapy for cervical cancer diagnosed by non-contact hysteroscopic technique

**DOI:** 10.3389/fmed.2025.1511052

**Published:** 2025-03-10

**Authors:** Xuemei Sun, Yinghua He, Xuedong Yang, Yu Wu, Yanhuan Yang, Yanping Wang, Xiuhua Fan

**Affiliations:** ^1^Changchun University of Chinese Medicine, Changchun, Jilin, China; ^2^Department of Proctology, Guang’anmen Hospital, China Academy of Traditional Chinese Medicine, Beijing, China; ^3^Department of Radiology, Guang’anmen Hospital, China Academy of Traditional Chinese Medicine, Beijing, China; ^4^Department of Gynecology, Guang’anmen Hospital, China Academy of Traditional Chinese Medicine, Beijing, China; ^5^Obstetrics and Gynecology Diagnosis and Treatment Center, The Affiliated Hospital, Changchun University of Chinese Medicine, Changchun, Jilin, China

**Keywords:** SVF, cervical cancer, chemoradiotherapy, non-contact hysteroscopic technique, auxiliary examination

## Abstract

Sigmoidovaginal fistula (SVF) is an extremely distressing and complex condition that significantly impacts a patient’s quality of life. The successful management of SVF relies on accurately identifying the fistula’s location and tract. However, preoperative localization can be challenging in certain cases. In this report, we describe a rare complication in a patient with stage IVA cervical cancer who developed SVF after concurrent chemoradiotherapy. Conventional diagnostic methods, including electron colonoscopy, methylene blue testing, and fistulography, were unable to locate the fistula. As an alternative, we used a non-contact hysteroscopic technique, which successfully identified the location, size, and number of fistulas. This method is particularly effective for patients with SVF, especially in postmenopausal women with narrowed or adherent vaginal tracts, women with intact hymen, and those with complex, high-grade vaginal fistulas resulting from cancer treatment with chemoradiotherapy.

## 1 Introduction

Sigmoidovaginal fistula (SVF) is a rare but serious complication after concurrent chemoradiotherapy (CCRT) for cervical cancer. Its symptoms are profoundly distressing, including the passage of flatus or stool through the vagina, local inflammation, irritation, and sexual dysfunction, all of which severely impact a patient’s quality of life (1, 2). Proper management of SVF requires accurate diagnosis and precise localization of the fistula to inform appropriate surgical interventions. However, in cases where fistulas develop after CCRT for cervical cancer, they are often located in the upper rectum and the fornix, above the level of the cervix. The complexity of the local anatomical structures at these sites makes it difficult to accurately identify and confirm the location, size, and number of fistulas.

The non-contact hysteroscopy technique, which does not require the use of a speculum, has become a preferred method for diagnosing and managing vaginal, cervical, and uterine disorders, especially in postmenopausal women with narrowed or adherent vaginal tracts and in women with intact hymen. In this report, we present a case of high-grade SVF that developed after chemoradiotherapy for stage IVA cervical cancer. After unsuccessful attempts to locate the fistula using electron colonoscopy, methylene blue test, and fistulography, we successfully used the non-contact hysteroscopy technique to determine the exact location, size, and number of fistulas, which facilitated the subsequent optimization of the surgical plan. We propose that non-contact hysteroscopy can serve as a valuable adjunct diagnostic tool for SVF, rectovaginal fistula (RVF), and similar conditions. Further investigation into its use is warranted.

## 2 Case report

**Figure 1:**
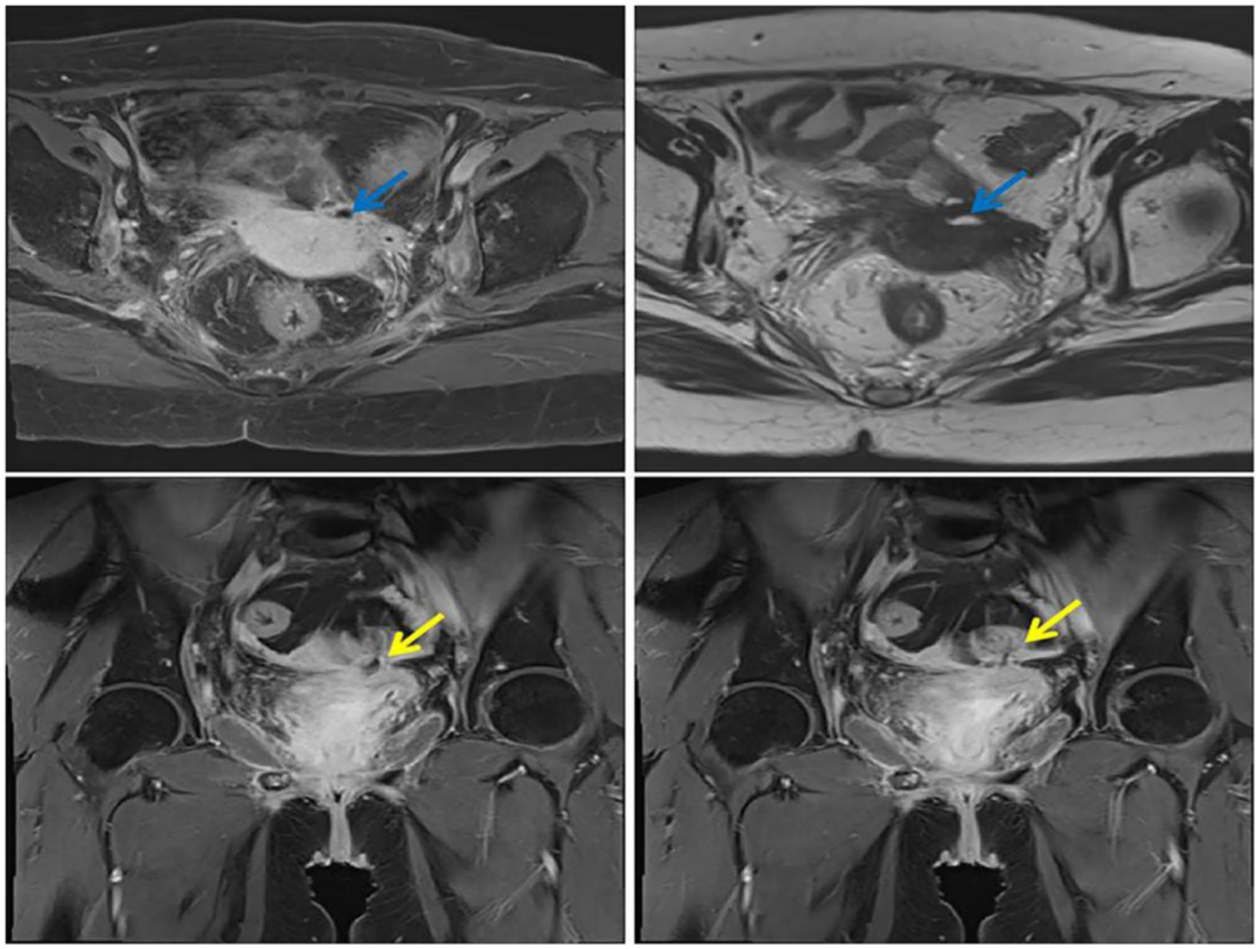
Pelvic MRI showing SVF (the blue arrow: axial Image; the yellow arrow: Coronal Image).

The patient is a 36-year-old female with a history of CCRT for stage IVA cervical cancer. She was admitted to Guang’anmen Hospital of China Academy of Chinese Medical Sciences on February 14, 2023, presenting with “vaginal flatus for 1 month and vaginal defecation for 3 weeks.” Pelvic magnetic resonance imaging (MRI) revealed the presence of an SVF (Figure 1). A methylene blue test showed a significant amount of blue-stained fluid emerging from the vagina, indicating a possible fistula in the left fornix. A barium enema revealed a tubular structure connecting the sigmoid colon to the nearby vaginal vault. An electronic colonoscopy showed anal access to the 20-cm sigmoid colon with repeated hooking and pulling; however, accessing the intestinal lumen was difficult, and no obvious fistula was observed. Gynecologic speculum examination revealed vaginal atrophy and narrowing, making it difficult to visualize the vagina and cervix directly. Based on these findings, the diagnosis of SVF was confirmed. Given the combined results of enteroscopy, barium enema, and other examinations, we determined that the patient’s fistula was located in a high position, surrounded by congested, edematous, and eroded mucosa. The local anatomical structure was complex and difficult to expose.

**Figure 2:**
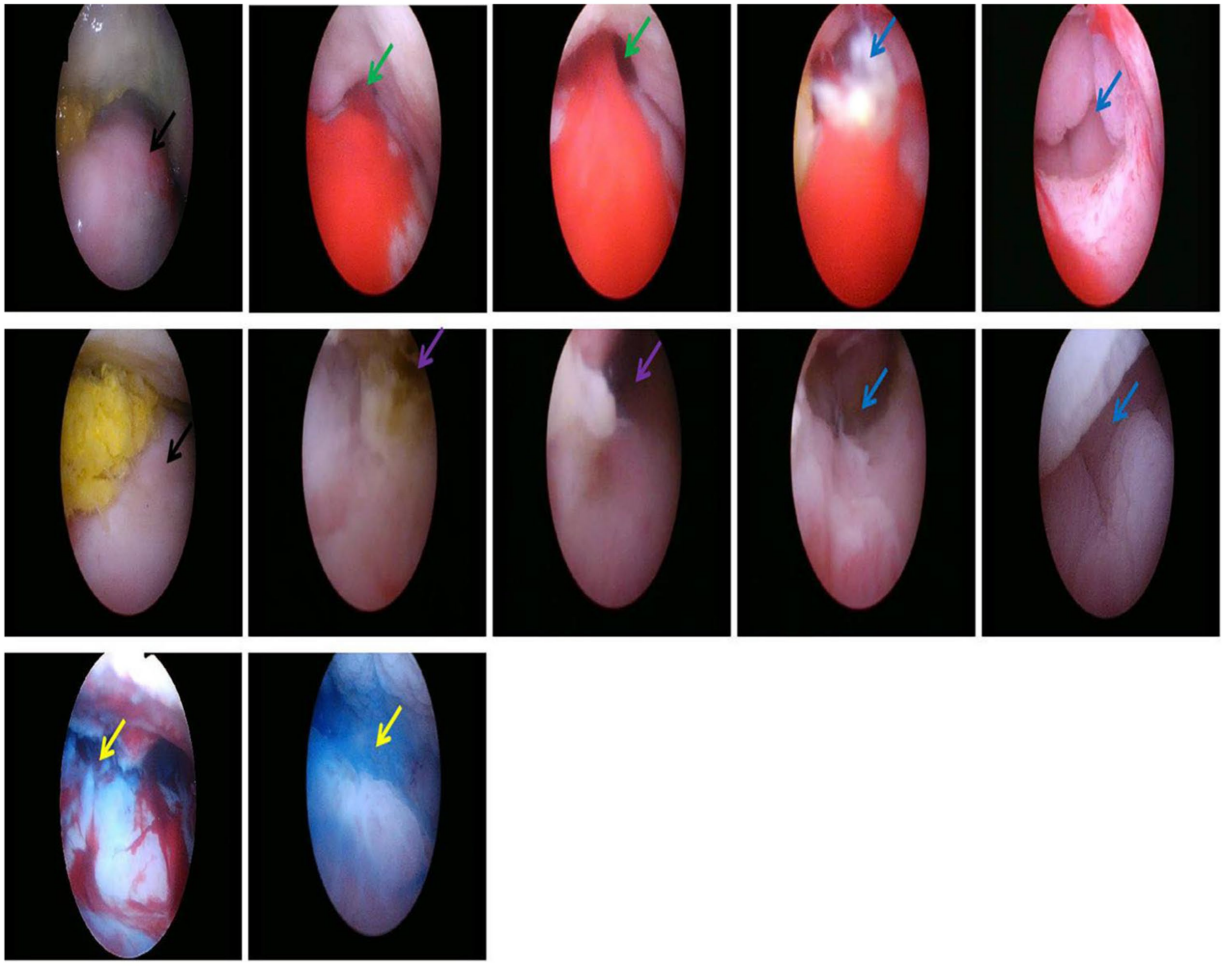
Uterine cervix (the black arrow), SVF in the left lateral fornix (the green arrow), SVF in the right lateral fornix (the purple arrow), Intestinal lumen (the blue arrow), Methylene blue (the yellow arrow).

To better localize the fistula, reduce the patient’s discomfort, and optimize the surgical repair plan, we used the non-contact hysteroscopy technique. The patient was placed in the lithotomy position, and the vulva and vagina were routinely disinfected with iodophor. Briefly, for patients with obvious vaginal atrophy who could not undergo routine disinfection, a 50-mL syringe was used to draw 0.5% of the iodophor solution and connected it to a disposable suction tube, which was inserted into the vagina from the external opening for flushing and disinfection. No vaginal speculum was used, and the hysteroscope was inserted directly through the vaginal opening, while the labia majora and minora were manually closed to create a sealed space, thereby minimizing the leakage of distension media and fully inflating the vagina. The vaginal wall, cervix, and fornix were observed sequentially. After confirming the fistula opening under direct vision, the size of the fistula opening and the surrounding tissues were observed for congestion, edema, necrosis, and feces, followed by entering the intestinal cavity to assess the fistulous tract and eliminate other intestinal lesions. Subsequently, methylene blue solution was injected through the anus, and a hysteroscope was placed through the vagina again. At this point, it was observed that a large amount of methylene blue solution overflowed from the fistula opening that could enter the intestinal cavity smoothly along the direction of the methylene blue solution. The examination revealed that the vaginal tract was patent, but atrophic. A small amount of pus-like debris was present around the upper part of the fistula. The mucosa appeared fragile and eroded, with fistulas visible in both the left and right fornices. Injection of melphalan fluid through the anus showed no resistance, and the diameter of the bilateral fistulas was approximately 2 cm, leading to the intestinal lumen. The cervix was significantly atrophic and was not well-visualized, and the hysteroscope did not enter the uterine cavity (Figure 2). The procedure was successful, with approximately 5 mL of intraoperative bleeding and 500 mL of rehydration fluid administered.

## 3 Discussion

SVF is an abnormal epithelialized connection between the sigmoid colon and the vagina. Common etiologies include birth trauma, diverticulosis, malignancy, Crohn’s disease, and surgical complications, with radiation exposure being a less frequent cause (incidence range: 0.3–6%) (3–5). The symptoms of SVF vary depending on the underlying cause, the location of the fistula, and the extent of the injury. Patients typically present with the passage of flatus or stool through the vagina, although more subtle symptoms such as vaginal discharge, sexual dysfunction, and systemic symptoms due to inflammation may also occur. These symptoms not only severely affect the patient’s quality of life but also pose a significant challenge in terms of social adaptation, making SVF an important concern in contemporary medicine.

Accurate localization of the fistula is crucial for determining the most appropriate surgical approach. Several diagnostic methods are available, including rectal palpation and vaginal-rectal double examination, vaginal speculum, rectal intraluminal and vaginal ultrasound, pelvic MRI, colonoscopy, the methylene blue test, fistulography, and vaginography. Digital rectal palpation and vaginal-rectal double examination revealed the course, height, and number of fistula orifices to assess the thickness of the perineal body, the tension of the anal sphincter, and the ability to control feces. However, it is challenging for fistulas with higher opening positions and smaller fistula orifices. Vaginal speculum examination is helpful, albeit its diagnostic value in identifying fistula openings is variable, with reported diagnostic rates ranging from 43 to 87% (6). The accuracy of this examination is limited by the lack of magnifying glasses, the difficulty of some patients in seeing the top of their vagina, and the difficulty of determining the exact location of the fistula in patients with feces present in the vagina. Rectal intraluminal and vaginal ultrasound are effective in identifying the fistulous trace (7, 8). Pelvic MRI offers superior visualization of muscle integrity surrounding the fistula and provides precise information regarding its location (9). Both these aspects have certain value in the diagnosis of SVF, but the localization of visible fistulas is often affected by various factors such as the size, location, and position of the fistula. Barium enema has been reported to identify only 30.8% of colovaginal and colovesicovaginal fistula cases (10). As recorded by Latchana et al., colonoscopy is a poor method for diagnosing colovaginal fistula, with a sensitivity of 12% in identifying colovaginal fistula (11). A colonoscopy helps observe the high SVF and evaluate the presence of inflammatory bowel disease, diverticulosis, and other intestinal lesions in the intestine. However, due to various factors, including small fistula orifices, location behind the folds of the colon, and curvature of the large intestine, the success rate of identifying fistulas remains low. The only way to diagnose with higher accuracy is an X-ray vaginogram, with an accuracy rate of 89% (11). Presently, no single method provides absolute accuracy in locating the fistula (11, 12). In most SVF cases, the fistula can be diagnosed and localized through a combination of physical examination, endoscopy, and imaging techniques. However, in some patients, diagnosis and localization are challenging due to factors such as the small size of the fistula, its high anatomical position, hidden location (e.g., behind colonic folds), and the complexity of the surrounding anatomy. Additional challenges include vaginal adhesions, stenosis, cervical atrophy, the presence of purulent discharge in the fornix, and narrowing of the intestinal lumen, along with brittle, congested, and edematous mucous membranes (6, 12).

The emergence of non-contact hysteroscopic technology fills a potential gap in the existing diagnostic protocols. Non-contact hysteroscopic technology is currently widely used in the diagnosis and treatment of abnormal uterine bleeding in adolescence (without destroying the intact hymen), postmenopausal vaginal fibro-epithelial polyps, and other diseases (13, 14). When compared with traditional hysteroscopy, without the use of vaginal speculum, the pulling and fixing of cervical forceps, and directly placing the hysteroscopy into the vagina and uterine cavity, it can significantly reduce the amount of distending fluid and operation time, as well as minimize the pain of patients. At the same time, it provides possibilities for the diagnosis and localization of SVF in women with malignant tumors undergoing pelvic radiotherapy and chemotherapy, vaginal stenosis or adhesions caused by menopause, and those with intact hymen. When compared with colonoscopy, non-contact hysteroscopy is performed through the vagina, which has several transverse folds and greater extensibility compared to that in the intestine, resulting in higher safety. The anterior wall of the vagina is 7-9-cm long and the posterior wall is 10-12-cm long. The anatomical structure is simple, the position is relatively fixed, the stability is strong, and it is easy to fully expand. Meanwhile, patients have long-term vaginal defecation, and the vagina is in a non-sterile environment. Preoperative vaginal iodophor washing and disinfection can effectively improve infection. However, colonoscopy involves exploration through the intestinal tract, which has poor flexibility and strong motility. Especially after chemotherapy and radiotherapy, local tissue congestion, ulceration, edema, or fibrous tissue hyperplasia can induce intestinal stenosis. This poses challenges for detecting small fistulas and hidden fistulas (located behind the folds of the colon). When compared with tandem vaginoscopy combined with colonoscopy, non-contact hysteroscopy does not require any flexible guide-wire insertion into the fistula opening of the vagina under endoscopic guidance nor does it require the endoscope to be introduced through the anus and advanced until the wire in the colon lumen is identified to confirm the fistula opening. Therefore, when compared to the traditional hysteroscopy, colonoscopy and tandem vaginoscopy combined with colonoscopy, non-contact hysteroscopy is simpler to operate, more comfortable, safe, and can be completed by gynecologists proficient in traditional hysteroscopy, making it more suitable for widespread clinical practice and promotion. When compared with auxiliary examinations such as barium enema and pelvic MRI, non-contact hysteroscopy magnifies the observation area through the scope, thereby allowing for comprehensive observation of the vagina, cervix, and fornix, which helps to detect fistulas in the deep and hidden areas of the vagina. It can not only accurately determine the location and numbers of fistula orifices under direct vision but also evaluate the size of fistula orifices, the trace of fistula, and the condition of local surrounding tissues, thereby eliminating intestinal lesions and providing assistance for subsequent management and optimal surgical approach selection. This approach offers all the advantages of visual localization of vaginal fistula orifices. However, due to the lack of a vaginal speculum and cervical forceps for traction and fixation, it is difficult to expose the top of the vagina and the vault. Notably, after inserting the hysteroscope through the external vaginal opening, the other hand should be used to close the labia majora and minora, close the vaginal opening, and close the water outlet of the hysteroscope, such that the vaginal wall bulges and forms a relatively closed and expanded cavity for clearer observation. Meanwhile, the non-contact hysteroscope body is relatively hard, warranting attention to gentle movements so as to avoid excessive and violent swinging movement of the body, which could reduce the occurrence of complications such as bleeding and tissue damage. Moreover, preoperative vaginal irrigation and disinfection with iodine and strict aseptic operation can reduce the occurrence of infection.

Presently, we have widely applied non-contact hysteroscopy technology in clinical practices for diseases such as SVF, RVF and vesicovaginal fistula (VVF). It is not only suitable for high-grade SVF, RVF and VVF after malignant tumor infiltration, invasion, or radiation therapy but also for inflammatory bowel diseases, birth trauma, infection, complications of gynecological and pelvic surgeries, and traumatic SVF, RVF and VVF, with broad applicability. At the same time, it provides possibilities for the diagnosis and localization of smaller fistulas, high-grade fistulas, and complex fistulas, as well as the diagnosis and treatment of vaginal fistulas induced by malignant tumor pelvic radiotherapy and chemotherapy, vaginal stenosis or adhesion after menopause, and intact hymen. Here, we exemplified high-grade SVF occurring after CCRT for stage IVA cervical cancer to explore the value of non-contact hysteroscopy technology. The patient in this case, a 36-year-old female with stage IVA cervical cancer, experienced vaginal flatus for 1 month and vaginal defecation for 3 weeks after completing CCRT more than a year prior. Based on her medical history, presenting symptoms, and supporting examinations, a diagnosis of SVF was established. For the follow-up management and determination of the optimal surgical plan, it was necessary to visualize and locate the fistula. Due to the high location of the fistula, poor intestinal flexibility, and strong motility, especially after chemoradiotherapy, the surrounding mucosa was congested, and edematous or fibrous tissue hyperplasia, complicated the colonoscopic examination. Repeated attempts to navigate the intestinal lumen with the colonoscope were challenging. Additionally, the patient’s vagina was atrophic and narrow, with congested mucosa near the cervix and the presence of a small amount of watery, fecal material. Attempts to use colonoscopy-mediated vaginal endoscopy to identify the fistula were unsuccessful. Ultimately, we utilized traditional hysteroscopy combined with non-contact hysteroscopic technology, which allowed for optimal distension of the vaginal canal and enhanced visualization of the cervix. This approach successfully localized the SVF, facilitating comprehensive preparation for subsequent surgical repair. We further proved the application value of non-contact hysteroscopic technology in the diagnosis and localization of SVF. Notably, the application of non-contact hysteroscopic techniques for diagnosing high-grade rectovaginal and colovaginal fistulas, particularly after CCRT, has not been documented previously. Thus, we demonstrated the diagnostic value of this technique in evaluating vaginal fistulas and to highlight the importance of multidisciplinary collaboration in such cases. This approach offers additional diagnostic options for the assessment and localization of vaginal fistulas. The present case patient had locally advanced cancer and a year after CCRT, he remained in the sequelae stage of CCRT. The local tissue blood supply was not abundant, the tissue quality was brittle after chemoradiotherapy, the risk of local infection was high, the failure rate of surgical repair and reconstruction were also high, and there was a possibility of peripheral tissue damage. After the final evaluation on February 24, 2023, the patient underwent “laparoscopic enterolysis and sigmoidostomy” under general anesthesia at our hospital and the operation went smoothly. On March 2, 2023, the patient reported that the stoma was unobstructed after surgery showed no other discomfort, and was allowed to leave the hospital. However, potential complications such as stoma necrosis, prolapse, and stenosis may occur after stoma surgery. Therefore, it is necessary to provide early health education to stoma patients, improve their self-care ability, and enhance their quality of life. The patient in question has been followed up online since discharge. On June 9, 2023, the patient reported smooth bowel movements, soft stool formation, no vaginal bleeding, no obvious discharge, and occasional small amounts of discharge from the anus. No vaginal, anal, or perineal discomfort was recorded. Regular follow-up of cervical cancer showed no recurrence. At the last follow-up (dated: February 22, 2025), the patient reported no discomfort and no recurrence of cervical cancer during routine follow-up.

## 4 Conclusion

The non-contact hysteroscopy technique employs the vagina as the surgical approach, which has large elasticity, simple anatomy, and strong stability. Meanwhile, it does not require the use of vaginal speculum and cervical forceps to fix the cervix, which minimizes the patient’s pain. With the magnifying effect of the lens, it is highly valuable for the diagnosis and localization of small and high-grade fistulas in SVF. Incorporating this technique into the existing diagnostic repertoire will prove particularly beneficial for postmenopausal women with narrowed or adherent vaginal tracts, women with intact hymen, and those presenting with high-grade complex SVF after chemoradiotherapy for cancer.

## Data Availability

The original contributions presented in the study are included in the article/Supplementary material, further inquiries can be directed to the corresponding authors.
